# Caregiver’s experiences with a mobile-based educational program and its impact on dietary treatment compliance of children with methylmalonic acidemia: an online survey

**DOI:** 10.1186/s13023-025-03528-3

**Published:** 2025-01-19

**Authors:** Qing Luo, Chunqin Liu, Lizhou Lin, Xuehua Liu, Huifang Chen

**Affiliations:** 1 School of Nursing, Guangzhou Medical University, Guangzhou, 510182 Guangdong China; 2 Operating Theatre, The First Affiliated Hospital of Sun Yat-sen University, Guangzhou, China; 3 Department of Hematology, Shandong Provincial Hospital Affiliated to Shandong First Medical University, Jinan, China

**Keywords:** Methylmalonic acidemia, Caregivers, WeChat public account, Dietary treatment compliance

## Abstract

**Background:**

Compliance to highly restrictive diets is critical for children with Methylmalonic Acidemia (MMA), and their caregivers play a prominent role in children’s dietary treatment from early childhood through to adulthood. Despite lots of efforts by the multidisciplinary medical team to ensure the smooth implementation of dietary treatment, restricting dietary protein remains particularly challenging for children with MMA. This study aimed to assess dietary treatment compliance in children with MMA and evaluate the impact of WeChat-based parent education on compliance.

**Methods:**

A sample of 151 caregiver-child dyads was obtained through online recruitment using convenience sampling from February to March 2023. At least one month following the enrollment of MMA caregivers in the WeChat public account “Methylmalonic Acidemia Diet Manager”, structured questionnaires were distributed to them through the electronic platform “Questionnaire Star” in collaboration with the Chinese National Alliance of Rare Diseases. Subsequently, the collected data was analyzed using quantitative methods.

**Results:**

Children with MMA aged over 5 years were more likely to present a lower level of dietary treatment compliance compared to those under 1 year old. Besides, the levels of children’s dietary treatment compliance were higher when their caregivers had higher levels of satisfaction and benefit from using the public account.

**Conclusion:**

Our findings highlighted the significance of age-related challenges in dietary treatment compliance among children with MMA and the promising impact of utilizing WeChat public accounts as a supportive education tool.

## Background

Methylmalonic Acidemia (MMA) is a rare and lethal inborn error of metabolism (IEM) that typically first with onset in infancy or early childhood, with a high mortality rate and poor long-term prognosis [2]. The condition is caused by mutations in the methylmalonyl-CoA mutase (MCM) gene or its cofactor, adenosylcobalamin (AdoCbl), leading to disruptions in the metabolic pathways of propionic acid precursor amino acids (valine, isoleucine, threonine, methionine), as well as propionate, odd-chain fatty acids, and cholesterol side chains. These disruptions result in the abnormal accumulation of 3-hydroxypropionic acid, methylcitric acid, and methylmalonic acid in blood and urine [11]. MMA is the most prevalent organic acidemia in children [21], and affected individuals often suffer from multi-organ damage, with potentially life-threatening consequences [28]. Despite advances in medical care, particularly the implementation of newborn screening, these benefits are mostly seen in short-term outcomes. However, over the course of the disease, recurrent episodes of metabolic acidosis and hyperammonemia remain persistent challenges [40]. Survivors often endure a range of chronic complications, including growth retardation, kidney failure, neurological dysfunction, and cardiomyopathy, among others [14, 16, 20, 40]. These complications frequently lead to early mortality, increased hospitalization, and a reduced quality of life.

*The guidelines for the diagnosis and management of PA and MMA* have recommended dietary and nutritional modifications aimed at reducing the frequency and duration of metabolic acidosis and hyperammonemic episodes [4]. Dietary management has become a core, lifelong therapy for MMA patients [33], involving the restriction of natural protein intake alongside supplementation with precursor-free medical foods. This approach aims to minimize the accumulation of toxic metabolites while ensuring adequate intake of essential amino acids, thereby promoting nutritional balance, metabolic stability, and optimal growth [1, 4, 33]. Studies have demonstrated that non-adherence to dietary recommendations could result in compromised therapeutic efficacy, prolonged hospitalizations, and increased burden for both patients and caregivers. In severe cases, non-compliance can accelerate disease progression and even result in death [6, 15]. In particular, unrestricted protein intake among MMA patients is a well-documented trigger for metabolic crises, placing non-compliant individuals at significantly higher risk of severe, potentially fatal complications [4]. Therefore, strict adherence to dietary treatments is crucial for managing MMA effectively.

However, achieving consistent compliance with this specialized dietary therapy remains a significant challenge, particularly for children. While multidisciplinary teams—including healthcare providers, dietitians, genetic counselors, and pharmacists—play a crucial role in integrating dietary management into the overall treatment strategy, their efforts are often hindered by persistent barriers. These teams are responsible for designing individualized dietary modifications, conducting regular check-ups, and providing ongoing health education, and they also implement crisis prevention protocols and coordinate referrals to specialists to manage the condition more holistically [11, 12, 37]. Despite these efforts, dietary protein restrictions pose particular difficulties. Family caregivers, particularly those major managing the care of children with MMA, are required to meticulously plan and adjust dietary intake based on medical advice and frequent hospital test results. Since this treatment is typically applied at home, family caregivers encounter difficulties in adhering to the prescribed regimen without direct professional supervision. Furthermore, medical foods prescribed for these patients often have poor palatability, leading to resistance, especially in children, who may experience symptoms such as vomiting, lethargy, and poor feeding [31]. These barriers significantly impact treatment compliance. Moreover, the high cost of these foods adds to the financial burden, further compounding issues of non-compliance. Despite continuous support and follow-up from healthcare teams, previous interventions have demonstrated limited success in fully addressing these compliance challenges.

The World Health Organization (WHO) describes treatment compliance as a complex dynamic process of individual behavior influenced by various factors, including social, economic, therapy-related, patient-related, situational, and health system-related elements [5]. These factors can be categorized as either internal or external to the patient. Undoubtedly, patients and their caregivers are the key part and final arbiters of a range of factors, and in the end, it is they who either do or do not follow the doctor’s advice and what is prescribed [30]. In addition, external factors such as improving taste or reducing medical foods prices to improve dietary treatment compliance cannot easily be modified in the short term. Thus, focusing on the internal factors is what’s most tangible and easiest to improve, which is also the most-studied area in clinical practice [30]. According to health belief model (HBM), the more patients and their caregivers are aware of their conditions and the significance of treatment, the more they can exert their subjective initiative related to health behavior, and the higher their compliance [32]. Thus, patients’ and their caregivers’ perception and correct understanding of the disease-related information is among one of the most important internal factors.

Recently, social media platforms have become a growingly popular source for health information and dissemination [39]. WeChat, one of the world’s largest mobile applications with 1.1 billion monthly active users, has gradually emerged as the most popular platform for health education in nursing management in China [38]. Studies have shown that WeChat-based health education programs can effectively improve patients’ health behavior, self-management ability, and treatment compliance in some diseases with similar situations in low treatment compliance, for example, in patients with cancer [17, 24] and chronic illness [10, 18]. Given these, WeChat-based health education is also likely to present new opportunities to facilitate dietary treatment compliance among children with MMA. Despite this promise, there remains a scarcity of research specifically investigating the role of WeChat-based health education in improving dietary treatment compliance in this patient population. To our knowledge, this research is one of the first attempts to use a WeChat-based platform to intervene in dietary compliance among children with MMA. The primary aim of our study was to fill this gap by exploring the potential effect of WeChat-based health education in supporting long-term dietary compliance among children with MMA. Through this research, we aim to identify effective strategies and interventions that could promote sustained adherence to dietary treatments, ultimately improving patient outcomes.

## Methods

### Study design and participants

This was an online cross-sectional study utilizing convenience sampling that was conducted between February and March 2023. The recruitment of caregiver-child dyads was supported by the cooperation with the staffs of the Chinese National Alliance of Rare Diseases (named the home of MMA and propionic acidemia). Then we created a WeChat group for MMA caregivers and guided them to follow the WeChat public account “Methylmalonic Acidemia Diet Manager”, ensuring that every caregiver could obtain health education information through the online WeChat platform. At least 1 month after the MMA caregivers followed the WeChat public account, the structured questionnaires administered by trained researchers were sent to caregivers by the Electronic “Questionnaire Star” platform (https://www.wjx.cn/) through the WeChat group. Reminder information was resent to non-responders 1 and 2 weeks after the initial investigation. The inclusion criteria of the MMA caregivers included: (1) one of the caregivers who provided major care for a child (aged < 18 years old) with a diagnosis of MMA confirmed by the diagnostic criteria and blood and urine chemistry, and the child’s condition was in a stable phase, (2) being 18 years of age or older, (3) having followed the WeChat public account “Methylmalonic Acidemia Diet Manager” for at least 1 month, (4) having the ability to use a smartphone or IPAD, and (5) expressing willingness to participate by signing an informed consent form. Participants with severe cognitive impairment, hearing impairment, communication disorders, or acute medical conditions were excluded. A total of 170 MMA caregivers were approached, out of which 19 participants who did not follow the WeChat public account were excluded. Ultimately, 151 responses were collected, resulting in a response rate of 88.8%. All procedures were in accordance with the ethics approval obtained from the ethics institutional review board at Guangzhou Medical University (No. L202212008). Written informed consent was obtained from all minors and their guardians or caregivers prior to participating in the study.

### Description of the WeChat public account

**Fig. 1:**
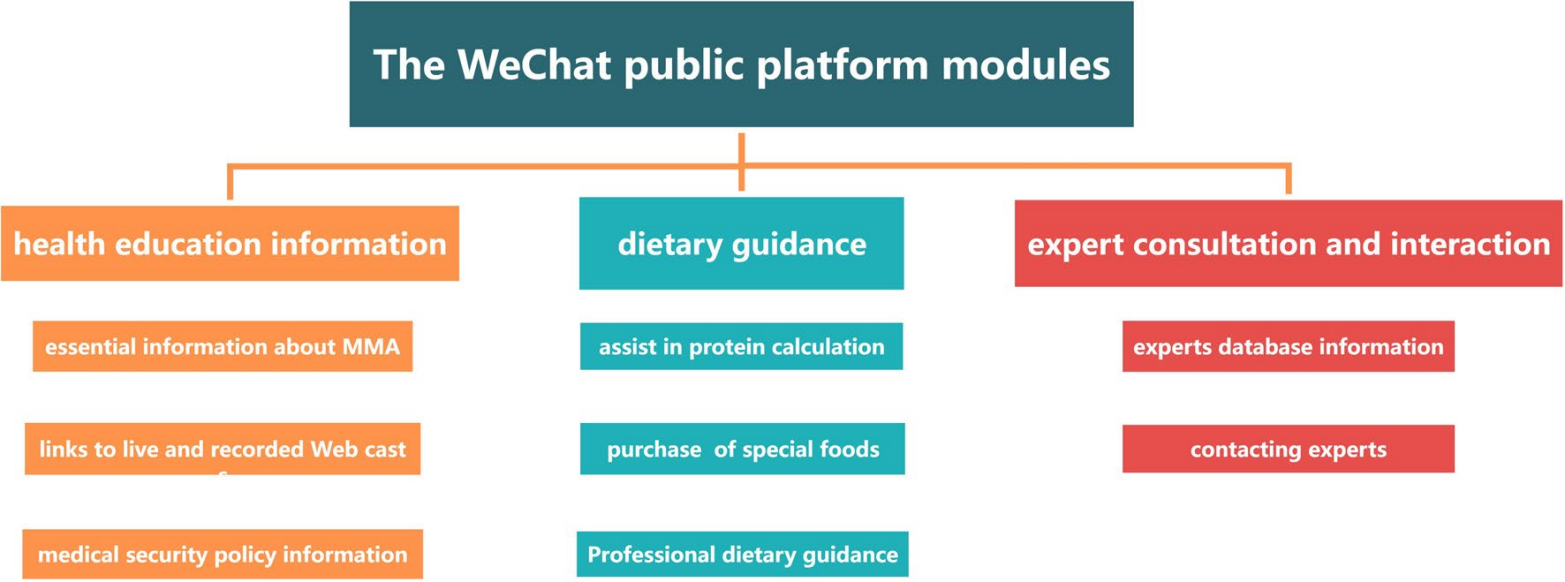
The WeChat public platform modules

The WeChat public account named “Methylmalonic Acidemia Diet Manager” was set up in 2022, aiming at providing diet and disease education for caregivers of children with MMA by multidisciplinary professionals such as pediatric specialists, nutritionists and nurses. The WeChat public platform was set up for three modules including health education information, dietary guidance, and expert consultation and interaction (see Fig. 1). (1) Health education information: once subscribed to the account, caregivers could access continuous online health education materials including essential information about MMA, links to live and recorded Webcasts of MMA-related scientific popularization activities, and medical security policy information. (2) Dietary guidance: MMA caregivers could also regularly obtain information on the basic principles of nutritional treatment and specific dietary advice, scientific feeding guidance, the complications that may occur, and the harms of going off the dietary treatment through online text, pictures, videos, animation and other forms. All of the information referred to up-to-date guidelines [11, 37], supplemented by high-quality studies, textbooks, and other trusted official sources [3, 25–27], helping to strengthen caregivers’ proficiency in dietary treatment skill application. (3) Expert consultation and interaction: the online multidisciplinary consultation expert team consisted of three pediatricians who are well-versed in rare diseases, a specialist pediatric nurse, a nutritionist, a social worker, and a physical rehabilitation therapist. The pediatricians provided professional treatment options and recommendations, the nurse provided professional nursing suggestions given the MMA children’s daily care problems, the nutritionist was responsible for nutritional counseling and guidance, and the social worker offered additional support such as medical assistance, psychological support, and social support. Besides, the physical rehabilitation therapist provided training program guidance for MMA children who have muscle tone abnormality or bradykinesia. MMA caregivers could access consult service at any time, and on the terminal of the cloud platform, the multidisciplinary team members would reply to them in time.

## Data collection

### Demographic, clinical, and dietary care characteristics of children with MMA

Demographic characteristics included age, gender, residence, and family annual income. Clinical variables included age of diagnosis, way of diagnosis, MMA genotype, and caregivers’ perceived disease severity. The dietary care status included the number of dietary treatment methods, daily duration of dietary care, and annual spending on dietary care. In addition, satisfaction with the dietary treatment was measured via one question, “Are you satisfied with your child’s dietary treatment?” (“dissatisfied”, “neutral” and “satisfied”), and the difficulty of dietary care was assessed by another question, “How difficult do you think your child is with dietary care?” (“difficult”, “neutral”, and “easy”).

### Caregiver’s use experience of the public account

This part primarily assessed the time after caregivers followed the public account, and the frequency of using the public account. Besides, the benefits, practicality, convenience, rationality, professionalism, and satisfaction of the public account were each assessed using a single question with a 5-level Likert scale from 1 (not at all) to 5 (extremely).

### MMA children’s dietary treatment compliance evaluation scale (MMA-DTCES)

Based on the theory of planned behavior (TPB), empirical literature, qualitative interviews, expert consultation, and psychometric test, the MMA-DTCES was developed in our previous studies [23]. It consists of 17 items and five domains: dietary treatment behavior attitude (3 items; one item of which, “I think the interruption of dietary treatment has no impact on my child’s condition”, is reverse scored), subjective norms (3 items), perceived control towards dietary treatment behavior(3 items; one item of which, “I think that long-term dietary treatment is difficult to accomplish”, is reverse scored), dietary treatment behavior intention(4 items), dietary treatment behavior(4 items). Respondents need to indicate the level of agreement on a five-point scale from 1 (strongly disagree) to 5(strongly agree). All the items are summed to provide a total score, with a score range of 17 to 85, and the higher scores suggest higher levels of dietary treatment compliance. In the present study, Cronbach’s alpha coefficient for the total MMA-DTCES was 0.874 and each dimension’s Cronbach’s alpha coefficient was 0.702 ~ 0.863, respectively.

### Data analysis

Data analysis was performed using SPSS 26.0. Means and standard deviations (SDs) were presented for continuous variables and frequencies and proportions (n, %) were presented for categorical variables. Independent t-tests and one way analysis of variance (ANOVA) were used to compare differences between groups. Multiple linear regression analysis was performed to identify the associate factors of dietary treatment compliance, and the *P* value for all tests less than 0.05 (two-tailed) was considered statistically significant.

## Results

### Descriptive statistics

A total of 151 children with MMA and their caregivers were analyzed in this study. Among these children, 86 (57.0%) were males, 34 (22.5%) were aged one year or less, and 48 (31.8%) were aged five years and above, with nearly half (49.7%) diagnosed before the age of 1 month. Two-thirds (67.5%) resided in Northern China. In terms of annual family income, the majority of families (60.9%) had an income of no more than 50,000 Chinese yuan, however, 94 (62.3%) of families’ annual spending on dietary care were over 20,000 Chinese yuan. More than one-third (39.1%) of caregivers spent more than 6 h per day on dietary care. Besides, 58.3% of the caregivers thought that their children had experienced severe symptoms, 22.5% were dissatisfied with the dietary treatment, and 64.9% thought it was difficult to implement dietary treatment. Other detailed demographic, clinical, and dietary care characteristics of children with MMA were presented in Table 1.

**Table 1:** Demographic, clinical, and dietary care characteristics of children with MMA (N = 151)

Variables	n (%)
Age (year)	
< 1	34(22.5)
1 ~ 5	69(45.7)
> 5	48(31.8)
Gender	
Female	65(43.0)
Male	86(57.0)
Current residence	
Northern China	102(67.5)
Southern China	49(32.5)
Annual family income (RMB)	
≤ 50,000	92(60.9)
50,001 ~ 100,000	41(27.2)
> 100,000	18(11.9)
Age of diagnosis (month)	
< 1	75(49.7)
1 ~ 6	50(33.1)
> 6	26(17.2)
The way of diagnosis	
Neonatal screening	69(45.7)
After acute stage onset	72(47.7)
Long-term symptoms	10(6.6)
MMA genotype	
MUT	114(75.5)
Others (eg., cblA, cblB)	37(24.5)
Number of dietary treatment method	
1	10(6.6)
2	39(25.8)
3	85(56.3)
4	17(11.3)
Number of caregivers	
1	76(50.3)
≥ 2	75(49.7)
Daily duration of dietary care (h)	
1 ~ 2	22(14.8)
2 ~ 4	31(20.5)
4 ~ 6	39(25.8)
> 6	59(39.1)
Annual spending on dietary care (RMB)	
< 10,000	17(11.3)
10,000 ~ 20,000	40(26.5)
> 20,000	94(62.3)
Caregivers’ perceived disease severity	
Slight	25(16.6)
Moderate	38(25.1)
Severe	88(58.3)
Satisfaction with the dietary treatment	
Dissatisfied	34(22.5)
Neutral	84(55.6)
Satisfied	33(21.9)
Difficulty of dietary care	
Difficult	98(64.9)
Neutral	44(29.1)
Easy	9(6.0)

RMB Chinese yuan

### Caregivers’ use experiences with the WeChat public account

66(43.7%) of caregivers have followed the public account for over 3 months and 40(26.5%) of them often use the public account. Concerning the caregivers’ point of view on the public account, the mean score of the benefits from using the public account, as well as the practicality, convenience, rationality, professionalism, and satisfaction of the public account were shown in Table 2.

**Table 2:** Caregivers’ use experiences with WeChat public account “methylmalonic acidemia diet manager”

Variables	n (%)/Mean (SD)
Time after following the public account (month)	
1 ~ 3	85(56.3)
> 3	66(43.7)
Frequency of using the public account	
Occasionally	44(29.1)
Sometimes	67 (44.4)
Often	40(26.5)
Benefits from using the public account	3.78(1.02)
Practicality of the public account	4.03(0.90)
Convenience of using the public account	4.02(0.89)
Rationality of the public account’s functional module	3.99(0.88)
Professionalism of the public account’s contents	4.08(0.80)
Satisfaction with the public account	4.07(0.81)

### Dietary treatment compliance

Descriptions of the MMA-DTCES were shown in Table 3, with a mean score of 65.66 (SD =  ± 9.11; range of 43 ~ 82). For the MMA-DTCES subscales, the dietary treatment behavior intention dimension had the highest mean score of 4.17(0.75), and the perceived control towards dietary treatment behavior dimension had the lowest mean score of 3.03(0.74).

**Table 3:** Descriptions of the dietary treatment compliance evaluation scale for children with MMA (MMA-DTCES)

Variables	Range	Mean	SD	Skewness	Kurtosis	Average scores of each dimension (M ± SD)
Dietary treatment compliance score	43–82	65.66	9.11	− 0.28	− 0.62	–
Dietary treatment behavior attitude	6–15	12.05	2.58	− 0.39	− 0.91	4.02 ± 0.86
Subjective norms	8–15	12.44	1.89	− 0.09	− 0.94	4.15 ± 0.63
Perceived control towards dietary treatment behavior	3–15	9.09	2.21	− 0.08	− 0.25	3.03 ± 0.74
Dietary treatment behavior intention	8–20	16.69	3.00	− 0.75	0.030	4.17 ± 0.75
Dietary treatment behavior	7–20	15.38	3.45	− 0.36	− 0.61	3.85 ± 0.86

### Univariate and multivariate analysis

Table 4 outlined the comparison of the mean score of dietary treatment compliance in different groups. Among these, there were significant differences concerning age, current residence, the age of diagnosis, and the frequency of using the public account (all *P* values < 0.05).

**Table 4:** The comparison of the mean score of dietary treatment compliance in different groups (N = 151)

Variables	Dietary treatment compliance M(SD)	*t/F*	*P*
Age (year)			
< 1	68.44(6.77)	39.626	**0.000**
1 ~ 5	69.72(7.02)		
> 5	57.83(8.31)		
Gender			
Female	65.40(9.06)	0.162	0.688
Male	66.00(9.24)		
Current residence			
Northern China	67.01(8.57)	7.231	**0.008**
Southern China	62.84(9.64)		
Family annual income (RMB)			
≤ 50,000	64.76(8.03)	1.123	0.328
50,001 ~ 100,000	67.50(9.47)		
> 100,000	65.66(9.11)		
Age of diagnosis (months)			
< 1	66.48(9.02)	3.783	**0.025**
1 ~ 6	66.70(9.61)		
> 6	61.27(7.23)		
The way of diagnosis			
Neonatal screening	67.36(8.89)	2.826	0.062
After acute stage onset	63.83(9.29)		
Long-term symptoms	67.00(7.44)		
MMA Genotyping			
MUT	65.63(9.55)	0.003	0.955
Others (eg., cblA, cblB)	65.73(7.72)		
Number of dietary treatment method			
1	65.70(8.82)	0.284	0.837
2	66.79(6.55)		
3	65.16(9.95)		
4	65.47(10.41)		
Number of caregivers			
1	64.72(9.94)	1.607	0.207
≥ 2	66.60(8.14)		
Daily duration of dietary care (h)			
1 ~ 2	63.14(8.08)	1.751	0.159
2 ~ 4	65.16(9.71)		
4 ~ 6	64.49(8.67)		
> 6	67.63(9.26)		
Annual spending on dietary care (RMB)			
< 10,000	65.34(9.01)	0.164	0.849
10,000 ~ 20,000	66.32(8.72)		
> 20,000	65.78(10.83)		
Perceived disease severity			
Slight	68.04(7.28)	0.828	0.439
Moderate	65.95(10.20)lePara>		
Severe	65.93(9.32)		
Satisfaction with the dietary treatment			
Dissatisfied	63.82(9.86)	1.553	0.215
Neutral	67.04(9.23)		
Satisfied	65.27(8.81)		
Difficulty of dietary care			
Difficult	65.41(9.52)	0.437	0.647
Neutral	66.82(9.45)		
Easy	67.22(9.32)		
Time after following the public account (month)			
1 ~ 3	64.92(9.21)	1.278	0.260
> 3	66.61(8.97)		
Frequency of using the public account			
Occasionally	62.91(9.92)	4.956	**0.008**
Sometimes	65.46(8.80)		
Constantly	69.00(7.72)		

Boldface indicates the signifcantly variables (*P* < 0.05)

Subsequently, only variables that were significantly associated with dietary treatment compliance in the univariate analysis and all the variables regarding caregivers’ comments about the public account were included in the multivariate linear regression analysis. Table 5 presented the results of multiple linear regression analysis (*R*^*2*^ = 0.556, adjusted *R*^*2*^ = 0.547, *F* = 61.291, *p* < 0.001). The findings revealed that the age of children served as a negative factor, while the satisfaction and benefits derived from using the public account were positive factors of dietary treatment compliance. Besides, the comparison of standardized coefficients indicated that the impact degree of the three significant independent variables related to dietary treatment compliance varied. However, it is important to interpret these coefficients cautiously, as they reflect relative influence rather than absolute importance. Specifically, with “1 year or younger” being used as the reference category, children aged over 5 years were more likely to present a lower level of dietary treatment compliance (*p* < 0.001). Besides, the levels of children’s dietary treatment compliance were higher when their caregivers had higher levels of satisfaction and benefit from using the public account (*p* < 0.05).

**Table 5:** Multiple linear analysis regression results

Factors	Unstandardized Coefficients (95%*CI*)	*SE*	Standardized coefficients	*t*	*P*	VIF
Constant	49.06(43.95, 54.17)	2.59	–	18.97	**0.000**	–
Satisfaction of using the public account	3.41(1.68, 5.14)	0.88	0.30	3.90	**0.000**	2.00
Benefits from using the public account	1.17(0.34, 3.09)	0.70	0.19	2.46	**0.015**	1.02
Practicality of the public account	− 0.93(− 3.38, 1.51)	1.24	− 0.09	− 0.76	0.452	4.88
Convenience of using the public account	1.58(− 1.02, 4.18)	1.31	0.16	1.20	0.231	5.38
Rationality of the public account functional module	0.53(− 2.35, 3.41)	1.46	0.05	0.36	0.717	6.39
Professionalism of the public account’s contents	0.29(− 2.75, 3.32)	1.53	0.03	0.19	0.852	6.00
Age(y)						
< 1	Reference					
1 ~ 5	1.354	1.35	0.07	1.00	0.319	1.80
> 5	− 11.86(− 13.98, − 9.74)	1.08	− 0.61	− 11.04	**0.000**	1.01
Current residence						
Northern China	Reference					
Southern China	1.48(− 0.83, 3.79)	1.17	0.08	1.27	0.207	1.18
Age of diagnosis(months)						
< 1	Reference					
1 ~ 6	1.88(− 0.47, 4.23)	1.19	0.10	1.59	0.115	1.23
> 6	0.63(− 2.47, 3.72)	1.57	0.03	0.40	0.689	1.38
Frequency of using the public account						
Occasionally	Reference					
Sometimes	− 0.70(− 3.28, 1.90)	1.31	− 0.04	− 0.53	0.598	1.68
Constantly	− 0.83(− 4.11, 2.44)	1.66	− 0.04	− 0.50	0.616	2.12

Boldface indicates the signifcantly variables (*P* < 0.05)

## Discussion

To our knowledge, this is the first study to explore the effect of using WeChat-based health education on children with MMA in terms of dietary treatment compliance in China. The current study fills a critical gap in the current literature by specifically examining the role of WeChat, a widely used mHealth platform in China, in supporting caregivers and enhancing treatment compliance. We found that the dietary treatment compliance of children was in an upper-medium level, in which the score of dietary treatment behavior intention dimension was the highest and the score of perceived control towards dietary treatment behavior dimension was the lowest. This suggested that children with MMA or their caregivers may have a strong willingness to adhere to dietary treatment but there were some difficulties and obstacles during actual implementation process, and further qualitative studies should be carried out to explore the reasons behind this phenomenon. In addition, our main finding was that the age of children with MMA, the caregivers’ satisfaction and benefit from using the public account were the major factors associated with children’s dietary treatment compliance.

In the current study, compared with children aged under 1 year, the school-aged children (aged over 5 years old) were more likely to present a lower level of dietary treatment compliance. Generally, preschoolers’ dietary management are considered the easiest to control as the content and amount of three daily meals are largely controlled by parents. Nevertheless, when children enter school and increasingly make his/her own dietary choices, it becomes a sensitive period to adapt and maintain a protein restriction diet. Studies have proved that in the transition period from childhood to adolescence, children with rare diseases would gradually lose social support and gain independence from experienced caregivers, which may be described as challenging for them due to the need of taking more self-management [9, 22]. Further corroboration can be found in a study that interviewed the barriers and facilitators of dietary treatment compliance among women with IEM, in which most participants reported they had difficulties with the diet treatment in the period of school. The reason may be that there are some difficulties existing in a new place including how to incorporate the diet into the school routine, the comments from peers and the temptation to go off dietary treatment [19]. This result indicated that we should pay particular attention to training affected children and their caregivers, as well as emphasizing the importance of treatment compliance in school-aged patients. Furthermore, early dietary education with both schoolmates and school officials may help improve long-term compliance.

Most importantly, the findings indicated that caregivers’ satisfaction and benefits of using the public account are significantly positively associated with MMA children’s dietary treatment compliance. While the unique features of WeChat make it particularly suitable for this Chinese context, the findings underscore the broader potential of tailored mHealth platforms in supporting caregivers and contributing to improved treatment compliance. However, it is important to note that the directionality of these associations should be interpreted with caution, and further researches, such as longitudinal study and randomized controlled trial, are needed to establish causal relationships. As a rare disease, MMA has the characteristics of small number of patients, rarity, low social understanding, low level of disease awareness, and very little media attention and public concern (von [35]). So that MMA patients often appeared to confront with many problems alone, for example, lack of understanding and knowledge about the disease [29], the inequality in access to new medical technologies and treatments [7], as well as lack of social support, professional assistance and reliable health education information [34], which impact the long-term treatment compliance. Thus, the patients and caregivers usually took responsibility for educating themselves by using the Internet to browse and locate disease information [13], but it should be aware that the quality of the information on the Internet may be inadequate and questionable [8]. Therefore, in the current study, the WeChat public account we established with three modules aimed to specifically address professional knowledge, dietary guidance and expert consultation about MMA, trying to serve as a sufficient and reliable source for the MMA patients and caregivers to obtain medical and health information. The results underlined that when MMA caregivers had higher levels of satisfaction and benefits from using the public account, their children would demonstrate higher levels of dietary treatment compliance. Due to the convenience, accessibility, and educational resources, WeChat public account may help realize information sharing between professionals and MMA caregivers, and enhance the level of personal health cognitive [36]. This can finally help them to effectively manage a variety of problems in the process of dietary treatment. Thus, further study is essential to examine the specific elements within the WeChat public account that drive higher satisfaction and perceived benefits among MMA caregivers. Elements such as accessible educational resources, interactive features, emotional support networks, and ease of navigation might significantly contribute to heightened satisfaction and perceived benefits. Besides, there is a need to explore how to optimize our WeChat public account to enhance support mechanisms and improve long-term compliance among children managing dietary treatments for MMA.

The main strengths of our study were its nationwide coverage. But several limitations should be acknowledged in interpreting our findings. The cross-sectional nature of our study precludes establishing causal relationships. In the future, longitudinal investigations tracking compliance over time and evaluating sustained effects of digital platforms on adherence are vital to guide future practice. And there is a need to conduct a WeChat-based intervention to provide clearer insights into the actual impact of WeChat-based health education on dietary treatment compliance of MMA caregivers in China. Additionally, other factors influencing dietary treatment compliance in MMA patients exist beyond those assessed in this study. For example, factors such as social network and psychological aspects, which have been shown to impact compliance in other chronic conditions, may also play a significant role in MMA. These factors should be explored in future studies to provide a more comprehensive understanding of dietary treatment compliance in MMA patients.

### Implications for practice

WeChat-based health education can be a convenient and accessible tool for spreading professional MMA-related health information, aiming at supporting MMA caregivers in dealing with common challenges. Notably, understanding the preferences and needs of caregivers regarding digital tools is crucial. Developing tailored content and resources on WeChat public accounts that offer practical tips, provide updated information, and foster peer interactions can significantly enhance engagement and treatment adherence. Thus, integrating WeChat public accounts or similar digital platforms into caregiver support programs will offer immense potential to enhance caregiver education, support, and treatment compliance for individuals with MMA.

## Conclusion

In conclusion, our findings highlight the significance of age-related challenges in dietary treatment compliance among children with MMA and the promising impact of utilizing WeChat public accounts as a supportive tool. Tailoring interventions to address age-specific challenges and leveraging digital platforms to provide accessible and beneficial resources could significantly improve the level of dietary treatment compliance. Developing WeChat-based health education has shown to be feasible and valuable, and further research is warranted to develop and evaluate comprehensive WeChat-based interventions aimed at enhancing compliance and improving the overall management of MMA.

## Data Availability

The data that support the findings of this study are available from the Chinese National Alliance of Rare Diseases, but restrictions apply to the availability of these data, which were used under license for the current study and so are not publicly available. The data are available from the corresponding author on reasonable request.

## Abbreviations

MMA: Methylmalonic acidemia
IEM: Inborn error of metabolism
MCM: Methylmalonyl-CoA mutase
AdoCbl: Adenosylcobalamin
WHO: World Health Organization
ANOVA: One way analysis of variance
MMA-DTCES: Dietary treatment compliance evaluation scale
HBM: Health belief model
TPB: The theory of planned behavior
